# Can we get out of the COVID pandemic without adequate vaccination coverage in the pediatric population?

**DOI:** 10.1186/s13052-022-01339-x

**Published:** 2022-08-19

**Authors:** Susanna Esposito, Rosanna Giordano, Giulia Paini, Matteo Puntoni, Nicola Principi, Caterina Caminiti

**Affiliations:** 1 Department of Medicine and Surgery, Pediatric Clinic, University of Parma, Via Gramsci 14, 43126 Parma, Italy; 2 Department of Public Health, AUSL Parma, Parma, Italy; 3 Research and Innovation Unit, University Hospital of Parma, Parma, Italy; 4 Università degli Studi di Milano, Milan, Italy

**Keywords:** COVID-19, COVID-19 vaccines, Epidemiology, Pediatric infectious diseases, SARS-CoV-2

## Abstract

**Background:**

During the first and second COVID-19 pandemic waves, children, despite susceptible to SARS-CoV-2 infection, appeared at lower risk of severe disease, hospitalization, and death than adults and the elderly. Moreover, they seemed to play a minor role in the diffusion of the virus. The aim of this manuscript is to show epidemiological surveillance on COVID-19 incidence and hospitalization in the pediatric cohort in order to explain the importance of an adequate COVID-19 vaccination coverage in the pediatric population.

**Methods:**

All subjects with documented SARS-CoV-2 infection diagnosed in Parma, Italy, between February 21st, 2020, and January, 31st, 2022, were recruited in this epidemiological surveillance. Diagnosis of infection was established in presence of at least one respiratory specimen positive for SARS-CoV-2 nucleic acid using a validated real-time reverse-transcriptase polymerase-chain-reaction (RT-PCR) assay.

**Results:**

The number of COVID-19 pediatric cases remained very low and lower than that recorded in the general population between early February 2020 and the end of October 2021, despite in the last part of this period the Delta variant emerged. On the contrary, starting from November 2021, a sharp and significant increase in COVID-19 incidence in the pediatric population was evidenced. This was detected in all the age groups, although greater in the populations aged 5–11 and 12–17 years old. Interestingly, the peak in hospitalization rate was observed in children < 5 years old, for whom COVID-19 vaccination is not approved yet. At the beginning of November 2021 among people older than 18 years of age 85.7% had completed the primary series of COVID-19 vaccine. Almost all the infants and pre-school children were susceptible. Until January 31st, 2022, 80.4% of adolescents aged 11–17 years had received at least two doses of COVID-19 vaccine and only 52.4% received the booster. Among children 5–11 years old, on January 31st, 2022, only 28.5% had received at least one vaccine dose.

**Conclusions:**

Compared with adults and the elderly, presently a greater proportion of children and adolescents is susceptible to SARS-CoV-2 and could play a relevant role for the prolongation of the COVID-19 pandemic. Only a rapid increase in vaccination coverage of the pediatric populations can effectively counter this problem.

## Introduction

During the first and second COVID-19 pandemic waves, children, despite susceptible to SARS-CoV-2 infection, appeared at lower risk of severe disease, hospitalization, and death than adults and the elderly [1–3]. Moreover, although they could transmit the infection, they seemed to play a minor role in the diffusion of the virus [4]. Along with the negative physical and mental impact, this was one of the most important reasons why many experts opposed the school closure as a measure to contain virus circulation [5–7]. With the extension of the pandemic, the emergence of viral variants and the development and use of effective and safe vaccines, the role of the pediatric population in the spread of SARS-CoV-2 has changed substantially. The aim of this manuscript is to show epidemiological surveillance on COVID-19 incidence and hospitalization in the pediatric cohort in order to explain the importance of an adequate COVID-19 vaccination coverage in the pediatric population.

## Methods

The province of Parma, a city with 194,417 inhabitants of whom 71,558 < 18 years of age, is located in Northern Italy and its health facilities were particularly involved in the first pandemic wave [8]. All subjects with documented SARS-CoV-2 infection diagnosed in Parma between February 21st, 2020, and January, 31st, 2022, were recruited in this epidemiological surveillance. Diagnosis of infection was established in presence of at least one respiratory specimen positive for SARS-CoV-2 nucleic acid using a validated real-time reverse-transcriptase polymerase-chain-reaction (RT-PCR) assay. Results of the specimens and on patients’ outcome were provided by the Department of Public Health of AUSL Parma, that collects all the results of subjects tested for SARS-CoV-2 using RT-PCR in the city.

## Results

**Fig. 1:**
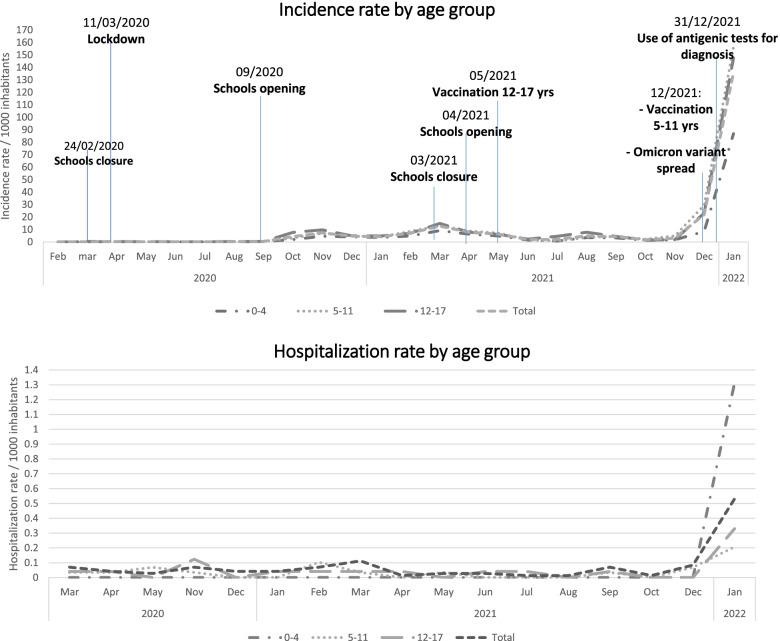
Surveillance on COVID-19 incidence (**A**) and hospitalization (**B**) in a pediatric cohort of 71,558 inhabitans

Figure 1 shows epidemiological surveillance on COVID-19 incidence and hospitalization in the pediatric cohort. The number of COVID-19 pediatric cases remained very low and lower than that recorded in the general population between early February 2020 (i.e., the onset of the pandemic) and the end of October 2021, despite in the last part of this period the Delta variant emerged. On the contrary, starting from November 2021, a sharp and significant increase in COVID-19 incidence in the pediatric population was evidenced. This was detected in all the age groups, although greater in the populations aged 5–11 and 12–17 years old. Interestingly, the peak in hospitalization rate was observed in children < 5 years old, for whom COVID-19 vaccination is not approved yet. Main reasons for hospitalization included diarrhea/vomiting (39%), respiratory distress/pneumonia (31%) and febrile seizures (11%).

## Discussion

Omicron SARS-CoV-2 variant has increased transmissibility than the original SARS-CoV-2 [9] and it cannot be excluded that, despite children were less susceptible than adults to the original virus [10], they can be more easily infected by the new virus. Moreover, when Omicron appeared, a great number of adults and elderlies had a certain degree of protection against this virus as many of them had been already infected or vaccinated against SARS-CoV2. At the beginning of November 2021 among people older than 18 years of age living in Parma 85.7% had completed the primary series of COVID-19 vaccine. On the contrary, children and adolescents with adequate protection were quite few. Almost all the infants and pre-school children were susceptible as very few had been previously infected and none of them was vaccinated. Vaccination of adolescents aged 11–17 years was introduced in May 2021: until January 31st, 2022, 80.4% of them had received at least two doses of COVID-19 vaccine and only 52.4% received the booster. Among children 5–11 years old, for whom vaccine was authorized in December 2021, on January 31st, 2022, only 28.5% had received at least one vaccine dose and coverage remained less than 35% up to March 30th, 2022.

## Conclusions

Compared with adults and the elderly, presently a greater proportion of children and adolescents is susceptible to SARS-CoV-2. Since, based on the global epidemiological data, health authorities currently tend to avoid further restrictive measures, it seems likely that children could play a relevant role for the prolongation of the COVID-19 pandemic. Only a rapid increase in vaccination coverage of the pediatric populations can effectively counter this problem. Every effort must be made to persuade parents who are uncertain or against COVID-19 vaccines. Moreover, an effective and safe vaccine for children < 5 years old must be quickly approved for emergency use.

## Data Availability

All data generated during this study are included in the published article.

## Abbreviation

RT-PCR: Real-time reverse-transcriptase polymerase-chain-reaction
